# Effect of repeated preheating on monomer elution from a bulk-fill composite resin

**DOI:** 10.34172/joddd.2023.40780

**Published:** 2023-12-30

**Authors:** Samet Atasoy, Serdar Akarsu

**Affiliations:** Department of Restorative Dentistry, Faculty of Dentistry, Ordu University, Ordu, Turkey

**Keywords:** Bulk-fill composite resin, Monomer elution, Preheated composite resin, Residual monomer

## Abstract

**Background.:**

Due to incomplete polymerization of composite resin restorations, residual monomers adversely affect their mechanical properties and biocompatibility. Preheating of composite resins is advised to increase the degree of conversion and reduce monomer elution. This study aimed to analyze the effect of preheating and repeated preheating on the amount of monomer released from a bulk-fill composite resin.

**Methods.:**

Forty samples were prepared using Filtek One Bulk Fill Restorative composite resin. Samples in one group were fabricated at room temperature, whereas the composite resins in the other groups were cured after 1, 10, or 20 repeated preheating cycles (55 °C), 10 in each group. Eluted urethane dimethacrylate (UDMA) and bisphenol-A-glycidylmethacrylate (BisGMA) monomers were measured with high-performance liquid chromatography (HPLC) 24 hours and 30 days after immersion. The data were evaluated using one-way ANOVA and post hoc Tukey tests. Paired-sample t tests were used to test the differences between time intervals.

**Results.:**

At both time intervals, the greatest amounts of released BisGMA, UDMA, and total monomers were obtained from the control group, whereas 10 preheating cycles resulted in the least monomer elution. The decrease in monomer elution was not statistically significant after 10 preheating cycles compared with that after one preheating cycle (*P*>0.05). The group with 20 preheating cycles showed a greater amount of monomer elution compared to that with 1 and 10 cycles, which was statistically significant (*P* < 0.05). The amount of released monomers on day 30 was significantly higher than on day 1 (*P*<0.01).

**Conclusion.:**

Preheating of the bulk-fill composite resin was shown to be effective in reducing monomer elution. However, monomer elution was adversely affected after repeated preheating cycles of 20.

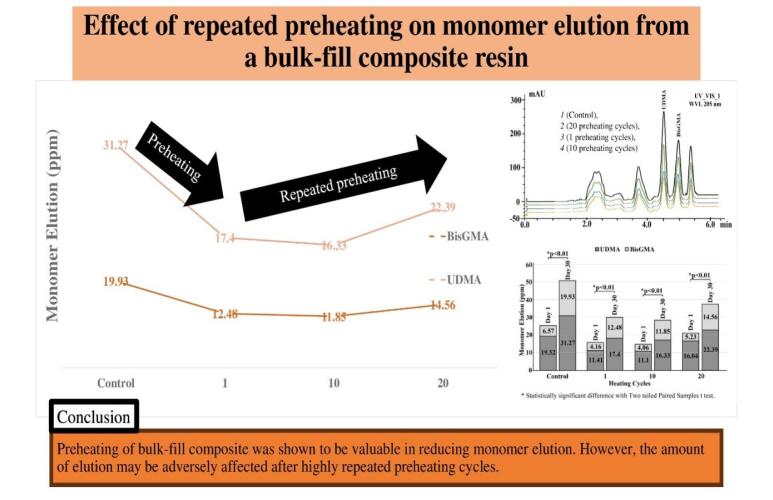

## Introduction

 Approximately 500 million direct dental restorations are performed worldwide annually, most of which are composite resin restorations.^1^ The success of composite resin restorations is attributed to their adhesive properties, resulting in minimally invasive preparation and reinforcement of the remaining dental structure.^2^ However, these materials are limited by polymerization-induced shrinkage and unreacted monomers that remain after polymerization.^3^

 Polymerization occurs in the organic matrix of dimethacrylate monomers, such as bisphenol A-glycidyl methacrylate (BisGMA) and urethane dimethacrylate (UDMA).^4^ Polymerization of monomers varies between 70% and 75%. The release of unpolymerized free monomers could induce biological responses such as mucosal irritation, pulp damage, and allergic reactions.^5^ Residual monomers could also lead to poor mechanical properties such as reduced hardness, wear resistance, and color stability.^6^ High-performance liquid chromatography (HPLC) is the most common analytic method used to determine the quality and the quantity of residual monomers.^7^

 Due to the limited polymerization depth of the conventional light activation process, incremental application of composite resins has been recommended, commonly in 2-mm-thick layers, to ensure sufficient polymerization and lower monomer elution.^8^ However, such a technique is clinically sensitive and time-consuming. To overcome these disadvantages, “bulk-fill” composite resins with a polymerization depth of 4 or even 5 mm and lower polymerization shrinkage than conventional composite resins have been introduced.^9^

 Another proposed alternative for optimizing the properties of dental materials is preheating. Some advantages reported in the literature with the preheating of composites before light polymerization include an increased degree of conversion,^10^ decreased elution of unreacted monomers,^11^ improved marginal adaptation of restorations due to reduced viscosity,^12^ and decreased polymerization-induced shrinkage stress.^13^ Furthermore, preheating also prevents temperature rise, which is unhealthy for the pulp, by reducing the duration of light curing.^14^

 However, most studies have analyzed the effect of preheating on the polymerization of composite resins, and studies analyzing the effect of repeated preheating cycles are limited. This information is important because the same composite syringe can undergo multiple clinical preheating cycles before being completely consumed.^15,16^

 This study aimed to analyze the effect of preheating and repeated preheating on the amount of monomer released from a bulk-fill composite resin for up to one month using HPLC. The null hypotheses tested were that the amount of monomer elution would not be affected by (1) preheating, (2) repeated preheating, or (3) different storage times.

## Methods

 A commercially available bulk-fill composite resin, Filtek One Bulk Fill Restorative, was used in this study. The specifications are presented in Table 1.

**Table 1:** Resin-based composite used in the present study

Material (Lot number)	Manufacturer	Type	Composition	Filler Mass/vol%
Filtek^TM^ One Bulk Fill Restorative (NA67764)	3M ESPE (St. Paul, MN, USA)	High viscosity	Aromatic urethane dimethacrylate (AUDMA), Urethane dimethacrylate (UDMA), dodecanediol dimethacrylate (DDMA), additional fragmentation monomer (AFM).	76.5/58.5

 The sample size was calculated using the G Power Software version 3.1.9.2 (Universität Düsseldorf, Germany) based on the previous sample size calculations. Forty samples were required in 4 groups, with a 95% confidence interval and 0.05 significance level.

**Figure 1:**
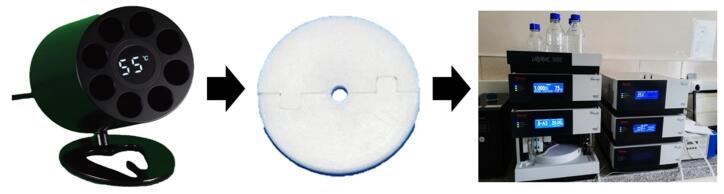
**(a)** Preheating of composites in a heating device ( AR Heat, Azdent, PRC) according to the number of repeated preheating cycles (Control, 1, 10, 20) **(b)** Preparation of samples with 4×5 mm polytetrafluoroethylene (PTFE) mould **(c)** Analysis of residual monomer amount with High Performance Liquid Chromatography (HPLC) (Dionex Ultimate 3000, Thermo Fisher Scientific, USA)

 One group of samples was fabricated at room temperature (RT) as a control group. In contrast, the composite resin samples in the other groups were cured after 1, 10, or 20 repeated preheating cycles (55 °C) in a preheating device (AR Heat, Azdent, PRC) (Figure 1).

 Preliminary tests were carried out to evaluate the heating and cooling times needed at RT (21±1 °C). Temperature variations in the composite resin were monitored using a digital multimeter equipped with a K-type thermocouple. A maximum of 10 minutes was required for the composite resin samples to reach a temperature of 55 °C. An equal amount of time was needed to return the composite resin samples to 21 °C. Each preheating cycle involved heating the composite resin samples for 10 minutes and then cooling it for another 10 minutes.

 Ten samples from each group were immediately packed into a cylindrical polytetrafluoroethylene (PTFE) mold with a height of 4 mm and an internal diameter of 5 mm (Figure 1). For all the tests, the average time between the composite resin removal from the heating device and light-curing was approximately 40 seconds. A mylar strip was placed between the glass slab and mold. After placing a sufficient amount of the composite resin in a capsule dispenser gun, the sample was covered with another Mylar strip to avoid contact with oxygen. The samples were then polymerized for 20 s at an intensity of 1200 mW/cm^2^ using an LED curing unit (Elipar S10, 3M ESPE, Seefeld, Germany). The light intensity of the LED curing unit was measured using a radiometer before and after curing. The tip of the curing unit was positioned centrally and in direct contact with the mold. After curing, the samples were carefully cleaned with a scalpel blade, and their thickness was measured using a digital caliper.

 Each sample was immersed in 1.5 mL of 75% ethanol solution and incubated at 37 °C. After 24 hours, the whole storage medium was removed for analysis. Afterward, the samples were air-dried and immersed in a fresh ethanol solution (1.5 mL). After retrieving from the storage medium, ethanol solutions were prepared and kept at 4 °C in the dark until the analysis. HPLC analysis was performed 24 hours and 30 days after immersion (Figure 1).

**Figure 2:**
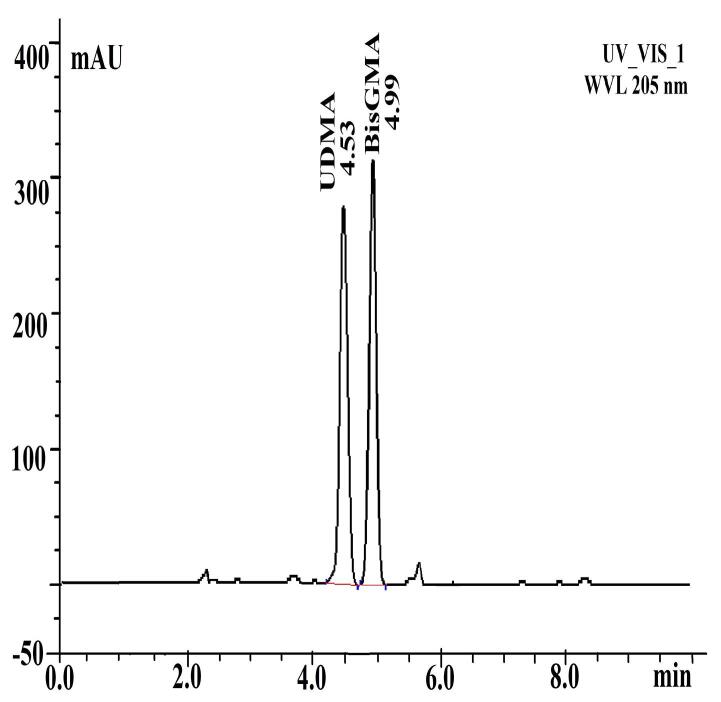
HPLC chromatogram of a mixture of reference monomers (BisGMA and UDMA)

 The solutions were analyzed with an HPLC instrument (Dionex UltiMate 3000, Thermo Fisher Scientific, Sunnyvale, CA, USA) equipped with a reverse-phase Thermo ODS Hypersil C18 column of 250×4.6 mm dimensions and 5-μm particle size. An isocratic method of acetonitrile/water (80:20) was used at a flow rate of 1 mL/min, and UV detection was set at 205 nm. The temperature of the column was 37 °C with a run time of 30 minutes for each sample. The residual monomers in the solutions were identified by comparing their retention times with those of the reference standards under the same HPLC conditions. BisGMA and UDMA standard solutions (Sigma Aldrich, St. Louis, MO, USA) had retention times of 4.99 and 4.53 min (Figure 2).

**Figure 3:**
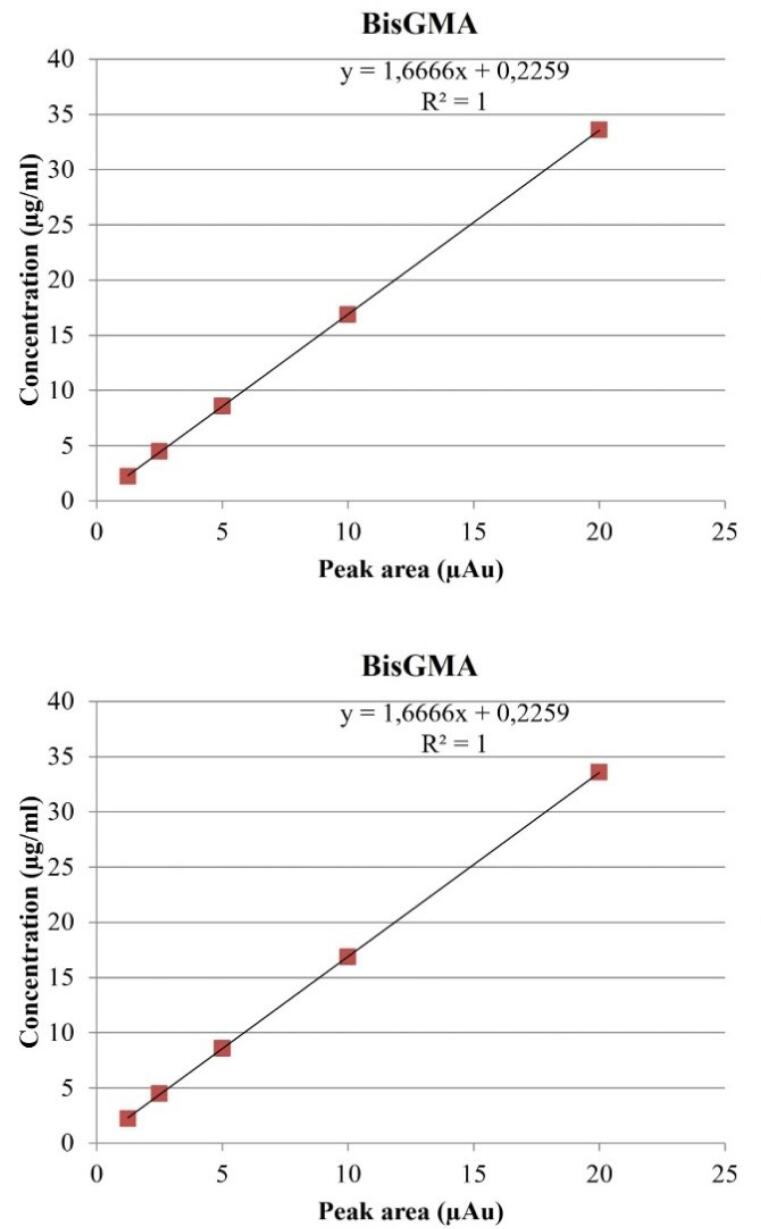
HPLC calibration curves for BisGMA and UDMA monomers

 A calibration curve was generated for each monomer standard using an external standard method (Figure 3). The monomer concentration was calculated using linear regression analysis of the values obtained from the calibration curve.

 Statistical analyses were performed using the SPSS software (v.26.0, Chicago, IL, USA). Differences in the eluted monomer concentrations between the study groups were evaluated using one-way ANOVA and post hoc Tukey tests. Paired-sample t-tests were used to analyze the differences between time intervals. The significance level was set at *P <*0.05.

## Results

**Figure 4:**
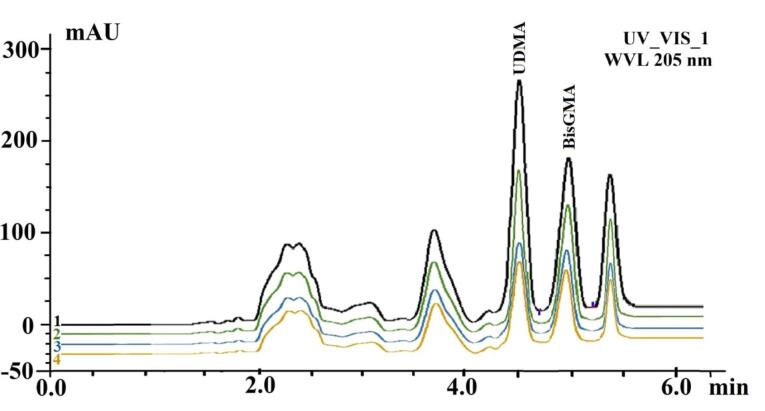
HPLC chromatogram with eluted monomers from different repeated preheating cycles.* 1* (Control),* 2* (20 preheating cycles),* 3* (1 preheating cycles),* 4* (10 preheating cycles)

Figure 4 shows the number of monomers eluted from different preheating cycles. The mean values (μg/mL) and the standard deviations (SD) of eluted UDMA, BisGMA, and total residual monomers for different repeated preheating cycles on days 1 and 30 are given in Table 2. The effects of the different preheating procedures on monomer elution were statistically significant (*P* < 0.001). In both time intervals, the greatest amounts of released BisGMA, UDMA, and total monomers were obtained from the control group, whereas 10 preheating cycles resulted in the least monomer elution. The decrease in monomer elution was not statistically significant after 10 preheating cycles compared with that after one preheating cycle (*P* > 0.05). The group with 20 preheating cycles showed a greater amount of monomer elution compared to that with 1 and 10 cycles and less monomer elution compared to the control group, which was statistically significant (*P* < 0.05).

**Table 2:** Comparison of study groups in terms of UDMA, BisGMA, and total residual monomer amount (μg/mL) on days 1 and 30

**Heating cycles**	**Day 1**			**Day 30**		
	**UDMA**	**BisGMA**	**Total**	**UDMA**	**BisGMA**	**Total**
Control	19.52 (2.87)^A^	6.57 (0.86)^A^	26.10 (3.70)^A^	31.27 (3.67)^A^	19.93 (2.39)^A^	51.21 (5.88)^A^
1	11.41 (1.74)^B^	4.16 (0.21)^B^	15.64 (2.30)^B^	17.40 (1.52)^B^	12.48 (0.65)^B^	30.08 (2.05)^B^
10	11.10 (1.98)^B^	4.06 (0.45)^B^	15.16 (2.40)^B^	16.33 (1.48)^B^	11.85 (0.86)^B^	28.29 (2.45)^B^
20	16.04 (1.29)^C^	5.23 (0.36)^C^	21.38 (1.21)^C^	22.39 (1.03)^C^	14.56 (0.69)^C^	36.86 (1.45)^C^
*P* value*	< 0.001	< 0.001	< 0.001	< 0.001	< 0.001	< 0.001

*Results of one-way ANOVA. For each monomer within a column, groups with different uppercase letter are significantly different (Tukey HSD test, *P* < 0.05).

**Figure 5:**
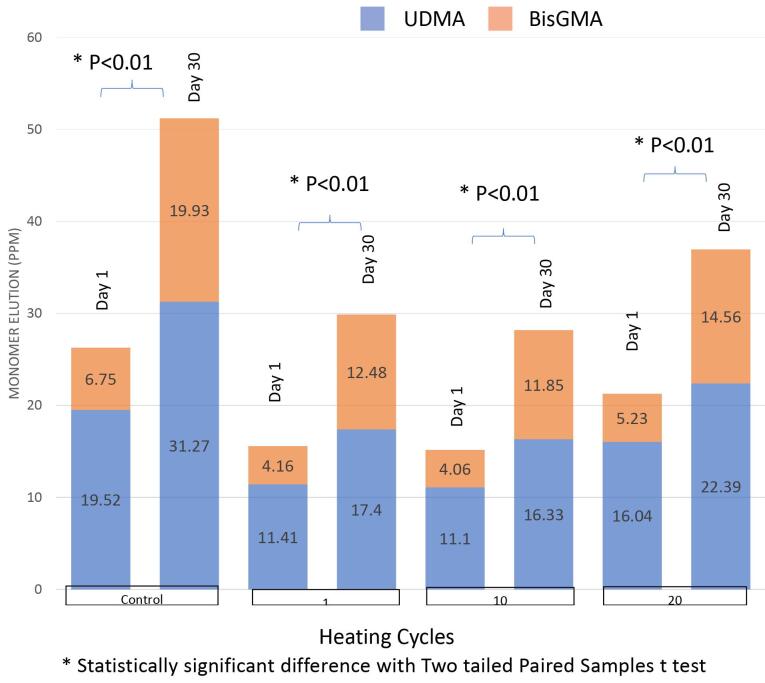
Comparison of days 1 and 30 in terms of UDMA, BisGMA, and total residual monomer (μg/mL)

Figure 5 presents mean values (μg/mL) of eluted monomer concentrations over time in each study group and the summary of statistical analysis showing significant differences between days 1 and 30 (*P* < 0.01). The highest amounts of released UDMA, BisGMA, and total residual monomers were observed after day 30.

## Discussion

 In this study, the effects of preheating and repeated preheating on the amount of monomer released from bulk-fill composite resins were evaluated by HPLC. The results showed that the amount of monomer elution was significantly affected by preheating, repeated preheating procedures, and storage time. Therefore, the null hypothesis was rejected.

 The mechanical and biological properties of composite resin restorations are highly correlated with their degree of polymerization.^17,18^ Numerous studies have shown that complete conversion of monomers is impossible, and only 70%–75% of monomers can be polymerized.^19^ Owing to incomplete polymerization, unreacted monomers will remain in the restoration, and about 10% of the remaining monomers may be released into the surrounding oral cavity, dentin, or pulp as a residual monomer when exposed to chemicals found in food and saliva.^20^ In this regard, the amount of residual monomer is a crucial parameter in determining the mechanical properties and biocompatibility of composite restorations.^21,22^

 Studies have reported that preheating composite resins reduces viscosity and increases monomer and radical mobility, resulting in a higher degree of conversion.^23,24^ Monomer elution decreases inversely with the degree of conversion.^25^ Unlike previous studies, a recent study found that preheating not only decreased the degree of conversion but also monomer elution of bulk-fill composite resins.^11^ Since bulk-fill composite resins do not always sustain homogeneous conversion, especially at 4-mm depth,^26^ detected double bonds may be stuck in the polymer structure and remain as pendant groups that are not free to be released.^19,27^ Therefore, the elution of unreacted monomers does not solely depend on the degree of conversion but is also related to other factors such as chemical structure of the polymer network and nature of the monomers.^7^

 The Filtek One Bulk Fill Restorative composite resin used in this study was a UDMA-based bulk-fill composite resin containing aromatic and aliphatic UDMA. In the present study, elution of BisGMA was also detected; even though it was not listed in the monomers specified by the manufacturer, UDMA elution was higher than that in previous studies.^28,29^ BisGMA exhibits high viscosity owing to its high molecular weight and strong intramolecular hydrogen bonding ability. However, the lower viscosity and higher double bond concentration of UDMA results in a high degree of conversion and crosslinking density.^27^ Sideridou and Achilias^7^ reported that the higher the crosslinking density, the higher the heterogeneity, and hence, the UDMA monomer elution is higher than that of BisGMA. In addition, neither AUDMA nor AFM was detected in the present study because their precise chemical structures are a trade secret, nor was 1, 12-dodecanediol dimethacrylate (DDDMA) detected because of the absence of a standard.

 In the present study, preheating of bulk-fill composites significantly reduced the amount of monomer elution, which is consistent with previous studies.^11,29^ Dunavári et al^29^ found that preheating significantly reduced the amount of UDMA and BisGMA monomer elution from Filtek One Bulk Fill Restorative composite resin, similar to the present study. In contrast, monomer elution increased when conventional composite resins were preheated. Bulk-fill composite resins can maintain an increased temperature for a relatively long time when applied at a 4-mm thickness compared to conventional composite resins at a 2-mm thickness.^30^ Additionally, the increased thermal storage capacity of high-viscosity bulk-fill composite resins, depending on their high filler content, may delay termination reactions of polymerization, leading to a higher amount of monomer elution.^31^ Moreover, Kincses et al^11^ showed that preheating reduced the amount of UDMA and BisGMA monomer elution from the Filtek One Bulk Fill Restorative composite resin, while it did not significantly reduce the amount of monomer elution from a different bulk-fill composite. Ebrahimi-Chaharom et al^32^ found that preheating slightly reduced the amount of monomer eluted from bulk-fill composite resins; however, this difference was not significant. This is because monomer elution is material-dependent.^11,29^ Another explanation is that monomer elution strongly depends on the different polymer network structures and the different monomer natures of various bulk-fill composite resins, as previously mentioned.

 No studies have evaluated the effect of repeated preheating cycles on the amount of residual monomers released from composite resins. Previous studies analyzing the effect of repeated preheating cycles on the polymerization process have found no correlation between 6 and 10 cycles of heating and the degree of conversion.^33,34^ However, the same composite resin syringe can be used for more than 20 restorations in clinical use before it is completely consumed, especially if it is recommended to be applied incrementally.^15,16^ Hence, 20 preheating cycles were included as the maximum number of repeated preheating cycles in the present study. The amount of monomer elution increased after 20 preheating cycles compared to 1 and 10 cycles. Previous studies have also shown that repeated preheating cycles may adversely affect mechanical and physical properties such as flexural strength after 20 preheating cycles^15^ and color stability after 40 preheating cycles.^35^

 Previous studies have shown that the monomer release increases over time.^7^ Hürmüzlü and Kılıç^36^ showed lower amounts of eluted monomers after day 1 and highest amounts after day 30. According to these findings, the highest amounts of eluted monomers were measured after day 30 compared to day 1 in the present study. However, in the present study, the increase in BisGMA elution on day 30 was greater than that of UDMA because BisGMA has a slow initial release due to its high molecular weight, and there is a greater increase in the subsequent release compared to that of UDMA.^7^

 In the present study, the amounts of the eluted monomers for different repeated preheating cycles were below the cytotoxic levels according to previous studies, except for that of UDMA elution, which was found to be 31.27 μg/mL on day 30.^37,38^ The amount of UDMA elution is above the level of cytotoxic effect on periodontal ligament fibroblast cells as reported by Geurtsen et al.^38^ However, because multiple composite resin restorations can be placed in the oral cavity, the number of eluted monomers should increase. In addition, in contrast to laboratory conditions, continuous saliva flow in the oral environment may affect the results for in vivoconditions. Furthermore, evaluating limited monomers is not a complete measure of the released components because other components, such as various monomers, initiator molecules, and fillers, are also shown in the chromatograms. Another limitation of this study is that only one bulk-fill composite resin was used, especially considering the strong material-dependent results.

## Conclusion

 Within the limitations of this study, the following conclusions can be reached:

1. Preheating a bulk-fill composite resin was shown to be valuable in reducing monomer elution.
2. However, the effects of repeated preheating cycles were different. Monomer elution was adversely affected after repeated preheating cycles of 20.
3. The best approach would be to use disposable composite capsules if preheating is preferred; further studies are necessary to confirm these results.

## Acknowledgments

 This study has been presented orally at the 26th Congress of the BaSS, May 2023, Skopje, Republic of North Macedonia. We would like to thank Ordu University, Central Research Laboratory and Lecturer Kubra Sueda AKINCI for their help in the HPLC analysis.

## Ethical Approval

 Not applicable.

## Competing Interests

 The authors declare that they have no competing interests.

## Funding

 This study was funded by Ordu University’s Scientific Research Coordination Unit (Project No: B-2102).
